# Acridone Derivatives for Near-UV Radical Polymerization: One-Component Type II vs. Multicomponent Behaviors

**DOI:** 10.3390/molecules29194715

**Published:** 2024-10-05

**Authors:** Adel Noon, Francesco Calogero, Andrea Gualandi, Hiba Hammoud, Tayssir Hamieh, Joumana Toufaily, Fabrice Morlet-Savary, Michael Schmitt, Pier Giorgio Cozzi, Jacques Lalevée

**Affiliations:** 1 CNRS, IS2M UMR 7361, Université de Haute-Alsace, F-68100 Mulhouse, France; adel.noon@uha.fr (A.N.); hammoudheba72@gmail.com (H.H.); fabrice.morlet-savary@uha.fr (F.M.-S.); michael.schmitt@uha.fr (M.S.); 2 Université de Strasbourg, F-67081 Strasbourg, France; 3 Laboratory of Materials, Catalysis, Environment and Analytical Methods (MCEMA), Faculty of Sciences, Doctoral School of Sciences and Technology (EDST), Lebanese University, Beirut 6573-14, Lebanon; t.hamieh@maastrichtuniversity.nl (T.H.); joumana.toufaily@ul.edu.lb (J.T.); 4 Department of Chemistry Giacomo Ciamician, University of Bologna, Via Selmi 2, 40126 Bologna, Italy; francesco.calogero2@unibo.it (F.C.); andrea.gualandi10@unibo.it (A.G.); 5 Center for Chemical Catalysis-C3, Alma Mater Studiorum-Università di Bologna, Via Selmi 2, 40126 Bologna, Italy; 6 Faculty of Science and Engineering, Maastricht University, P.O. Box 616, 6200 MD Maastricht, The Netherlands

**Keywords:** free radical polymerization, photoinitiators, visible range, light-emitting diodes, 3D patterns

## Abstract

In this work, two novel acridone-based photoinitiators were designed and synthesized for the free radical polymerization of acrylates with a light-emitting diode emitting at 405 nm. These acridone derivatives were employed as mono-component Type II photoinitiators and as multicomponent photoinitiating systems in the presence of an iodonium salt or an amine synergist (EDB) in which they achieved excellent polymerization initiating abilities and high final conversions of the acrylate group. Photoinitiation mechanisms through which reactive species are produced were investigated employing different complementary techniques including steady-state photolysis, steady-state fluorescence, cyclic voltammetry, UV–visible absorption spectroscopy, and electron spin resonance spectroscopy. Finally, these molecules were also used in the direct laser writing process for the fabrication of 3D objects.

## 1. Introduction

Acridones and their derivatives are considered as recognized molecules in the scientific community owing to their highly fluorescent properties and excellent stability against photodegradation, oxidation, and heat [1,2]. These compounds have been deployed as fluorescent labels for amino acids [3], peptides [4], substrates for catalysis [5], and for the preparation of highly photoresistant and selective chemosensors [6], and they were also used as new DNA probes for DNA detection [7]. Moreover, the acridone molecule contains an electron-accepting carbonyl group and an electron-donating amino group, which is a contributive property for the formation of a photoexcited intramolecular charge transfer state, thereby enhancing the electron transfer rates [8,9]. In this context, acridone derivatives were utilized as photocatalysts to mediate the visible light-induced organocatalyzed atom transfer radical polymerization (ATRP) of vinyl monomers [10] and the photo-induced direct arylation of arenes and heteroarenes with aryl diazonium salts [11].

Free radical photopolymerization is considered nowadays a green technology that is used in diverse applications. This technique offers many advantages over traditional thermal polymerization techniques including fast polymerization rates, high spatial and temporal control, low energy consumption, and preparation of solvent-free formulations [12,13], and thus it has been advanced successfully to be used in different industrial applications comprising adhesives, coatings, 3D printing, and electronics [14,15,16,17,18]. Type II photoinitiators (PIs), well-known PIs in activating the free radical polymerization (FRP) process, are often used [12]. They generate initiating radicals under light irradiation through electron and proton transfer processes with another co-initiator component [17]. The majority of commercial PIs used in this field have absorption properties in the 200-360 nm range, which necessitates using UV exposure as an irradiation source [19,20], whereas recent efforts are focusing on the development of photoinitiating systems that absorb at longer wavelengths (near-UV to visible range), taking advantage of deeper penetration and the use of safe, simple, cheap, environmentally friendly, and low power consumption light-emitting diodes (LEDs) as irradiation sources [21,22].

**Scheme 1:**
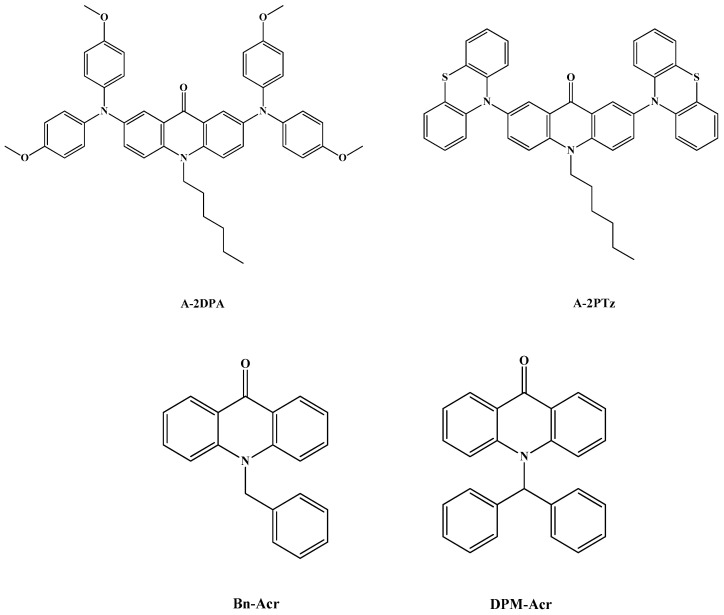
Chemical structures and abbreviations of acridone derivatives: previously studied (A-2DPA, A-2PTz) in [26], and the newly investigated ones in this work (Bn-Acr and DPM-Acr).

In previous work, acridone was used along with diphenyliodonium chloride for the FRP of methyl methacrylate (MMA) under UV exposure [23]. In another prior work, two photoinitiating systems including the combination of acridone with chlorine (acridone-Cl_2_) or bromine (acridone-Br_2_) were designed for the FRP of MMA under visible light [24,25]. Nevertheless, these former studies demonstrated some limitations in the polymerization conditions including irradiation by UV light [23], a heating temperature reaching 40 °C, and exploitation of CCl_4_ as a potently toxic solvent [24,25]. In order to activate the FRP of (meth)acrylates and the cationic polymerization of epoxides following exposure to the irradiation of a laser diode at 405 nm, Lalevée and colleagues have recently created two acridone derivatives (Scheme 1) and employed them in the presence of iodonium salt or/and amine [26]. In this work, two new mono-component Type II photoinitiating systems designated as Bn-Acr and DPM-Acr (Scheme 1) have been synthesized and utilized for the FRP of di(trimethylolpropane)tetraacrylate (TA). These PIs were also used in parallel as photosensitizers to sensitize an iodonium salt (Iod) or an amine synergist (EDB). The resulting two-component photoinitiating systems were then used for the FRP of TA with increased polymerization rates. The photoinitiation capabilities of the above-mentioned suggested systems were examined when they were exposed to an LED at 405 nm, and the mechanisms controlling the photoinitiation process were studied using various approaches and characterization techniques. Moreover, one of these photoinitiating systems was potentially used in laser writing applications for the fabrication of 3D patterns as proof of its high photoreactivity.

## 2. Results and Discussion

### 2.1. Synthesis of Bn-Acr and DPM-Acr

**Scheme 2:**
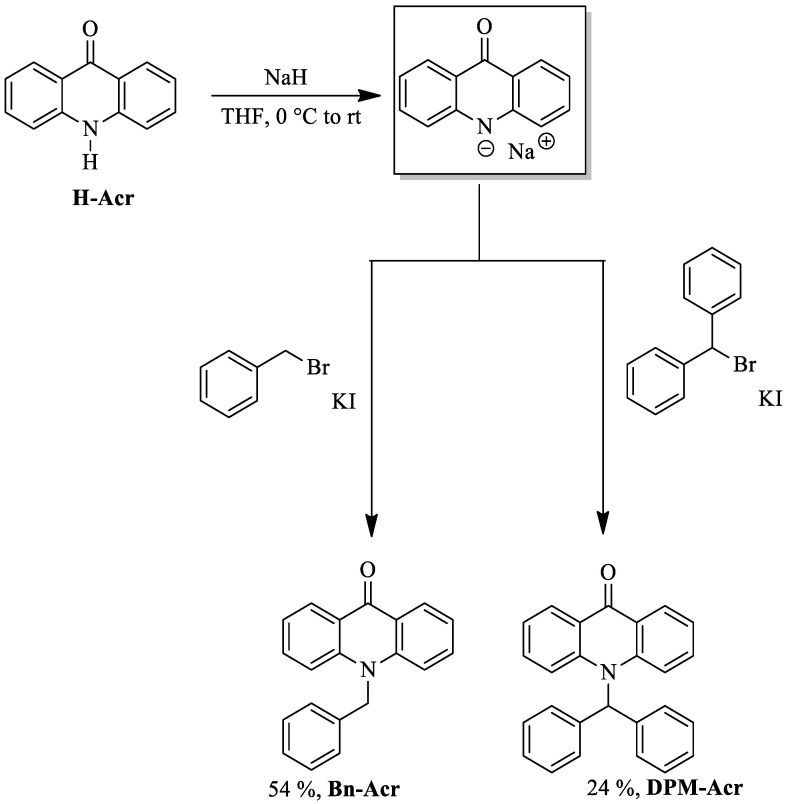
Synthesis of the target PIs.

The synthetic strategy employed for the synthesis of the target PIs (Scheme 2) is based on previously reported methodologies for the N-functionalization of variously decorated acridones and step-by-step described including the characterizations within the supporting information [27]. Sodium hydride was found to be the most effective base for the deprotonation of commercially available Acr-H and the formation of the corresponding intermediate Acr^−^Na^+^. The target products Bn-Acr and DPM-Acr were obtained with moderate yields after electrophilic quenching of Acr^−^Na^+^ by benzyl bromide or benzhydryl bromide, respectively. Notably, to further enhance the reaction outcome, in both cases, a substoichiometric amount of potassium iodide was added to the reaction medium. This addition has proved to be beneficial to the reaction as it facilitates the transient formation of the corresponding iodide [28]. The NMR properties of the synthesized acridone compounds are as follows: Bn-Acr: 1H NMR (600 MHz, CDCl3) δ 8.61 (d, J = 8.0 Hz, 2H), 7.67–7.61 (m, 2H), 7.39–7.28 (m, 7H), 7.22 (d, J = 7.5 Hz, 2H), 5.61 (s, 1H). 13C NMR (151 MHz, CDCl3) δ 178.64, 142.97, 135.86, 134.43, 129.62, 128.19, 126.02, 123.00, 122.02, 115.55, 51.21; DPM-Acr: 1H NMR (600 MHz, CDCl3) δ 8.52 (dd, J = 8.0, 1.8 Hz, 2H), 7.43–7.38 (m, 2H), 7.38–7.30 (m, 7H), 7.29–7.27 (m, 6H), 7.24–7.17 (m, 2H). 13C NMR (151 MHz, CDCl3) δ 179.01, 143.24, 137.76, 132.93, 128.95, 128.32, 128.10, 127.46, 123.87, 121.76, 118.04, 67.58 (see more information and NMR data in the Supplementary Information).

### 2.2. UV–Visible Absorption Properties

**Figure 1:**
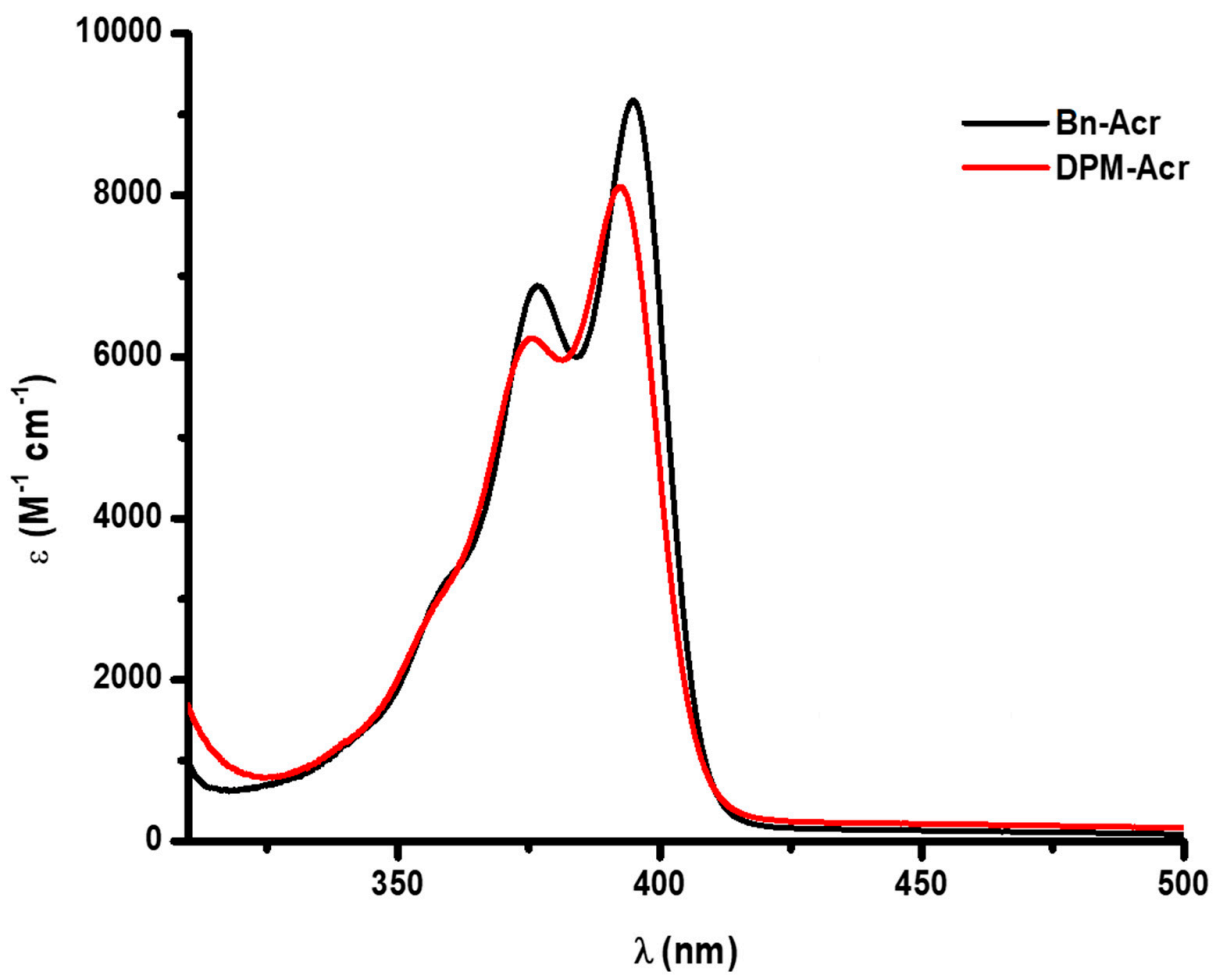
UV–visible absorption properties of compounds Bn-Acr and DPM-Acr in acetonitrile.

The newly established acridone-based PIs optical absorption spectra in acetonitrile are shown in Figure 1, and Table 1 compiles the related data (maximum absorption wavelengths (λ_max_), molar extinction coefficients at λ_max_ (ε_max_), and the emission wavelength at 405 nm (ε_405nm_)). The emission spectra of the near-UV and visible LEDs employed in this work are compatible with the good absorption properties that the two PIs demonstrated in the 325–415 nm range. The maximum absorption wavelengths (λ_max_) of the two PIs are nearly the same with 395 nm and 376 nm for Bn-Acr and 392 nm and 375 nm for DPM-Acr. Remarkably, both PIs showed high molar extinction coefficients at their λ_max_ and at λ = 405 nm, which is related to the π-electron delocalization property attained due to the presence of one benzene ring in Bn-Acr and two benzene rings in DPM-Acr.

### 2.3. Free Radical Polymerization (FRP) of Acrylates

**Figure 2:**
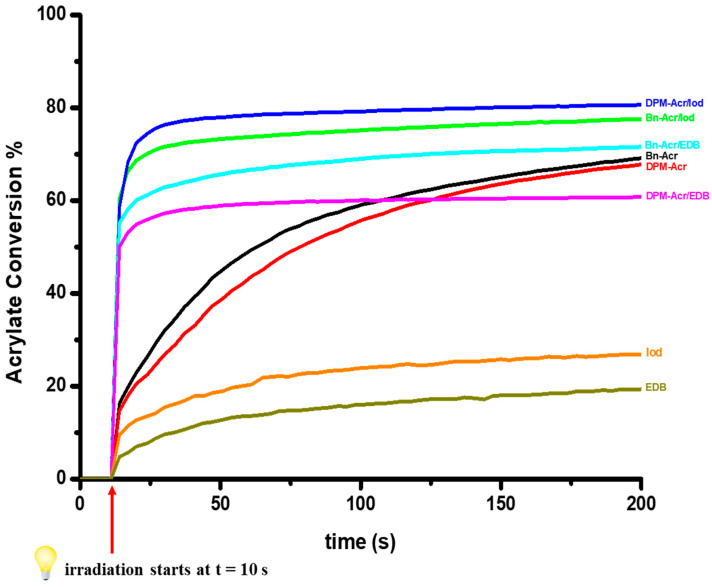
Photopolymerization profiles of TA (acrylate function conversion vs. irradiation time) in laminate (thickness = 25 μm) upon exposure to an LED (λ = 405 nm) in the presence of PIs (1% *w*/*w*), PIs/Iod (1%/1% *w*/*w*), PIs/EDB (1%/1% *w*/*w*), Iod alone (1% *w*/*w*), and EDB alone (1% *w*/*w*). The irradiation starts at t = 10 s.

The photoinitiation abilities of acridone-based compounds in the polymerization of TA-acrylate monomer were studied using RT-FTIR in thin films (25 μm, in laminate) upon irradiation with LED@405 nm. These compounds acted as one-component Type II PIs (1% *w*/*w*) and as multicomponent PI systems in the presence of Iod as an additive (1%/1% *w*/*w*) or in the presence of EDB as an additive (1%/1% *w*/*w*). Figure 2 displays acrylate function conversion vs. irradiation duration profiles, and Table 2 provides a summary of the corresponding final acrylate function conversions (FCs).

**Scheme 3:**
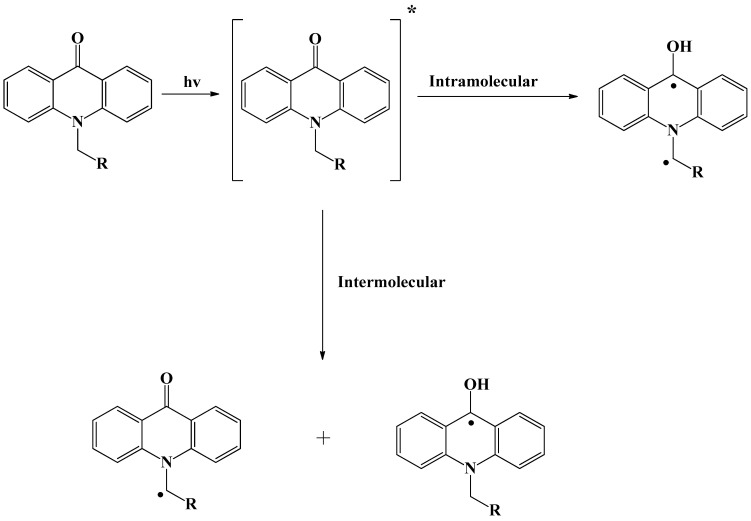
The proposed photochemical mechanism for acridone-based PIs as one-component Type II PIs.

The obtained results showed that both compounds (Bn-Acr and DPM-Acr) functioning as one-component Type II PIs were able to initiate the FRP of TA without the addition of a co-initiator where they achieved relatively high acrylate function conversions (69% and 68%, respectively) with moderate polymerization rates. It is known that Type II PIs require a co-initiator to effectively generate free radicals which justifies the observed attenuated behavior in the polymerization rates, whereas these acridone-based PIs can undergo intramolecular or intermolecular proton transfer reactions to generate initiating radicals (more details are mentioned in the photochemical mechanisms section; Scheme 3). Moreover, we observed that the addition of an iodonium salt has a favorable impact on the final acrylate function conversions and the rates of polymerization compared to those obtained with PIs alone, where the FC increases from 69% to 77% for Bn-Acr and from 68% to 81% for DPM-Acr and the rate of polymerization becomes faster for both systems (the maximum acrylate conversion is reached from the first few seconds of irradiation). The same positive effect on the rates of polymerization was also noticed after the addition of the amine synergist (EDB) to the acridone-based PIs, where we detected rapid polymerization rates compared to the PIs alone, but not on the final acrylate function conversion where it stayed nearly the same for Bn-Acr and decreased slightly for DPM-Acr. This difference in the impact of both co-initiators on the FCs of the acrylate group could be related to stronger electron transfer reactions that occurred between Bn-Acr and DPM-Acr with iodonium salt (more details are mentioned in the photochemical mechanisms section; Equations (r1–r4)). In addition, it is significant to mention that Iod or EDB tested separately could not initiate the FRP of TA upon exposure to an LED@405 nm where low FCs were observed (26% and 19%, respectively), evidently demonstrating the importance of the combination of acridone-based PIs with the mentioned additives for an efficient process. Additionally, these multi-component PI systems were also studied using RT-FTIR in thick films (2.3 mm, under air) upon the same irradiation for the FRP of TA (Figure S1), and they achieved high FCs of the acrylate group (68% and 82% for Bn-Acr and DPM-Acr with Iod, respectively, and 83% and 77% for Bn-Acr and DPM-Acr with EDB, respectively) where tacky-free polymers were also obtained.

### 2.4. Photochemical Mechanisms

#### 2.4.1. Steady-State Photolysis

**Figure 3:**
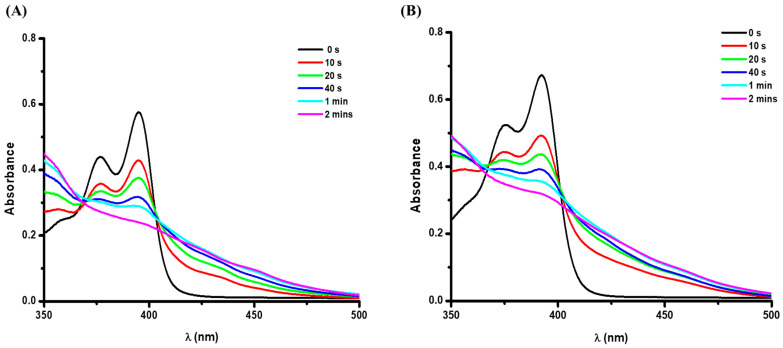
Photolysis of (**A**) Bn-Acr with Iod (10^−2^ M) and (**B**) DPM-Acr with Iod (10^−2^ M) in acetonitrile using LED at λ = 385 nm.

Steady-state photolysis experiments of PIs alone and with Iod (10^−2^ M) or EDB (10^−2^ M) were performed in acetonitrile under light irradiation (LED@385 nm). Bn-Acr and DPM-Acr have shown no photolysis occurring which could be attributed to the low concentration of PIs used to perform these experiments, and rather the concentration of the PIs must be relatively high for them to be acting as efficient one-component Type II PIs (intermolecular reaction). On the other hand, Figure 3A,B show that we observed a fast photolysis behavior and isosbestic points appearing for both PIs in the presence of Iod. This indicates that the investigated PIs in their excited states had a favorable interaction with the iodonium salt and that photochemical processes occurred without side reactions. These findings are consistent with the high photoinitiation abilities as shown in the photopolymerization experiments. In addition, the photolysis experiments of both PIs in the presence of EDB displayed a slight change in the original absorption spectra after 2 min of irradiation (Figure S2A,B), which could be linked to the fact that the photoproducts obtained were not in the absorption range we were observing.

#### 2.4.2. Fluorescence Quenching and Cyclic Voltammetry

**Figure 4:**
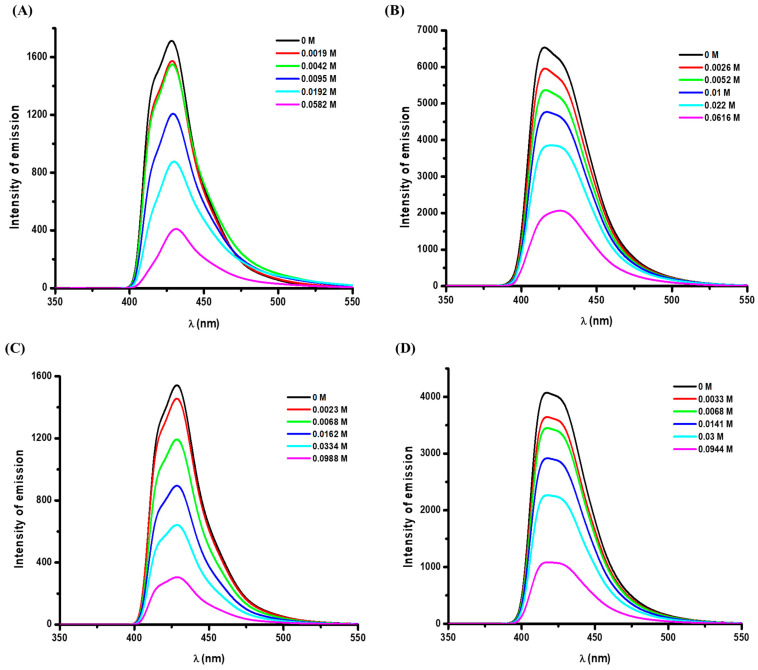
Fluorescence quenching study of Bn-Acr (**A**) and DPM-Acr (**B**) by Iod in acetonitrile; fluorescence quenching study of Bn-Acr (**C**) and DPM-Acr (**D**) by EDB in acetonitrile.

To construct an enhanced comprehension of the interaction between our newly investigated acridone PIs and the co-initiators (Iod and EDB), fluorescence quenching experiments were carried out in acetonitrile. The concentrations of Iod and EDB were elevated progressively and each time a fluorescence spectrum was procured. Figure 4A,B show a prompt fluorescence quenching occurring for both Bn-Acr and DPM-Acr by Iod, respectively. The same behavior was also observed for both PIs by EDB (Figure 4C,D), which attests to the strong interaction of our PIs with both Iod and EDB (as shown in Equations (r1–r4) below). In addition, the associated Stern–Volmer plots (Figure S3A–D) allow us to calculate the Stern–Volmer coefficients (K_sv_) and the electron transfer quantum yields (Φ_et_), which are gathered in Table 3. High electron transfer quantum yields were obtained (76% and 68% for Bn-Acr and DPM-Acr with Iod, respectively; 80% and 74% for Bn-Acr and DPM-Acr with EDB, respectively), which also verifies the strong PI/additive interaction.

The oxidation and reduction potentials (E_ox_ and E_red_) determined by cyclic voltammetry (Figure S4A,B), together with the singlet excited state energy ES1 (determined from the crossing point of the absorption and fluorescence spectra; Figure S4C,D), enabled the evaluation of the free energy change (ΔG) for the electron transfer reaction between both PIs and the additives (Iod and EDB) (Table 3). Highly favorable ΔG is found following the strong PI/Iod and strong PI/EDB interactions observed in the fluorescence quenching experiments.

#### 2.4.3. ESR Experiments

In the aim of thoroughly examining the photoinitiation mechanisms of acridone-based PIs acting as mono-component Type II PIs or as multicomponent photoinitiating systems, ESR spin trapping experiments of Bn-Acr alone, Bn-Acr/Iod, and Bn-Acr/EDB were performed upon irradiation with LED@405 nm.

**Figure 5:**
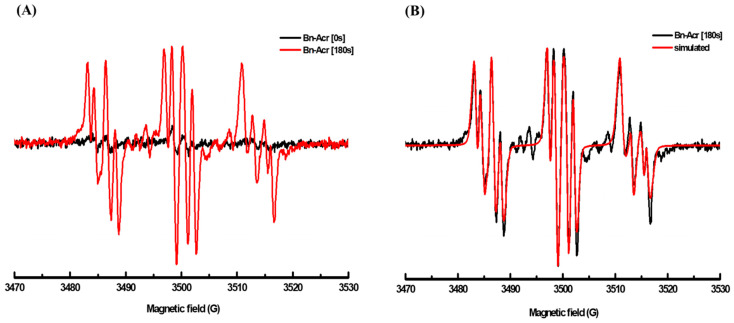
ESR spectra for Bn-Acr recorded in the presence of PBN and tert-butylbenzene with an LED@405nm: (**A**) before and after irradiation; (**B**) experimental and simulated spectra observed after irradiation (at t = 180 s).

Radicals adjacent to the amine group were trapped by PBN for the Bn-Acr photolysis after 180 s of irradiation (Figure 5). Values of the hyperfine coupling constants for the detected radical adduct were α_N_ = 13.9 G, α_H1_ = 1.4 G, and α_H2_ = 3.5 G, which could be assigned to the radicals close to the amine group (Acr-CH^●^-R). Therefore, according to all the obtained former results and based on the literature [12,17], we can say that the ketone group in this one-component Type II initiating system was excited by light irradiation and reached an excited state. Then, the hydrogen abstraction of the amine unit in the excited molecule could proceed through intramolecular or intermolecular proton transfer reactions to produce radicals near the amine group that could start polymerization reactions, as well as ketyl radicals from the ketone group that are ineffective in starting the polymerization reactions. The details of the proposed main mechanism leading to possible initiating radicals related to acridone-based PIs acting as one-component Type II PIs are illustrated in Scheme 3.

**Figure 6:**
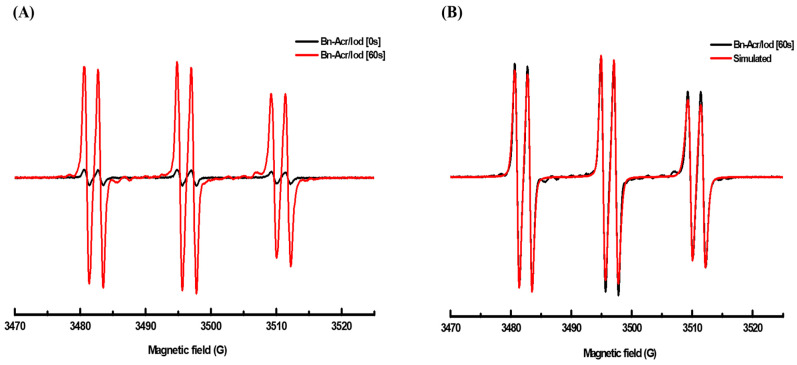
ESR spectra for Bn-Acr/Iod recorded in the presence of PBN and tert-butylbenzene with an LED@405nm: (**A**) before and after irradiation; (**B**) experimental and simulated spectra observed after irradiation (at t = 60 s).

Furthermore, after 60 s of radiation, PBN trapped aryl radicals in the Bn-Acr/Iod solution (Figure 6). The obtained radical adduct had hyperfine coupling constant values of α_N_ = 14.3 G and α_H_ = 2.1 G. These values can be attributed to the aryl radical (Ar^●^), which is formed through a photo-oxidation process in which an electron is transferred from the excited state of the PI (^1^Acr) to Iod (Equations (r1) and (r2)). These radicals are recognized as the initiating species for the FRP. In addition, PBN in Bn-Acr/EDB solution revealed aminoalkyl radicals following 180 s of radiation (Figure S5). The radical adduct obtained had values of α_N_ = 14.3 G and α_H_ = 2.1 G as hyperfine coupling constants. These values can be attributed to the aminoalkyl radicals (EDB^●^(–H)) that are responsible for starting the FRP process. They are formed by a photo-reduction process where an electron is transferred from EDB to the excited state of the PI (^1^Acr) (Equation (r3)) followed by a hydrogen abstraction (Equation (r4).

Acr (hυ) → ^1^Acr (r1)

^1^Acr + Ar_2_I^+^ → Acr^●+^ + Ar_2_I^●^ → Acr^●+^ + Ar^●^ + ArI (r2)

^1^Acr + EDB → Acr^●−^ + EDB^●+^ (r3)

Acr^●−^ + EDB^●+^→ Acr-H^●^ + EDB^●^ (−H) (r4)

### 2.5. Direct Laser Write (DLW) Experiments

**Figure 7:**
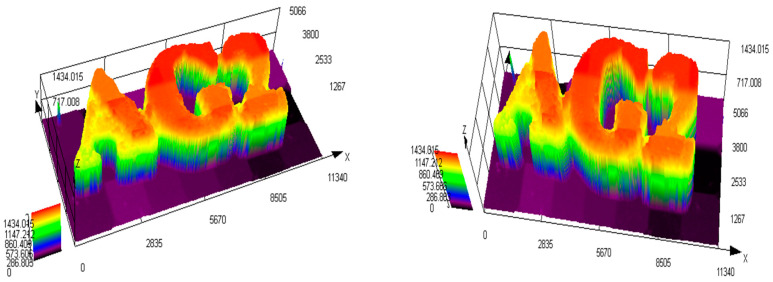
Three-dimensional patterns for compound DPM-Acr with Iod (0.1%/1% *w*/*w*) in TA were produced after exposure to a laser diode at 405 nm and were analyzed by numerical microscopy.

The two-component system DPM-Acr/Iod (0.1%/1% *w*/*w*) was chosen to carry out the direct laser write (DLW) tests because of its notable photoinitiation capabilities in the FRP of TA among the investigated PI systems. DLW was used to obtain the denotation “AG2” displayed in Figure 7 using a laser diode at 405 nm. Numerical optical microscopy was used to describe the denotation. Remarkably, the created 3D pattern has a high spatial resolution (polymerization process occurred only in the irradiated area), it has the following dimensions (width ≈ 11340 µm, height ≈ 5066 µm, thickness ≈ 1430 µm), and only requires 30 s of irradiation time to generate, confirming the system’s strong photosensitivity (DPM-Acr/Iod).

## 3. Experimental Part

### 3.1. Synthesis of the Investigated Acridone-Based PIs

The synthetic routes employed in the direction of getting the newly investigated molecules (Bn-Acr and DPM-Acr) are given above in Section 2.1. In addition, the detailed experimental description of this synthetic procedure along with the characterization of these compounds are provided in the supporting information.

### 3.2. Other Chemical Compounds

**Scheme 4:**
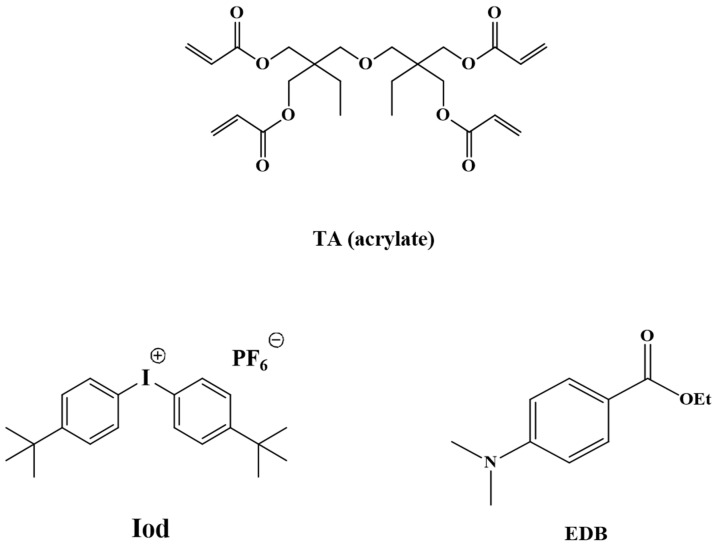
Chemical structures and abbreviations of the benchmark monomer TA, and the additives (Iod) and (EDB).

The chemical structures of the chemical compounds that were used to prepare the resin formulations are displayed in Scheme 4. These compounds were chosen with the highest purity available and used exactly as received. The monomer used (TA) was obtained from Sartomer-Europe. The storage stabilizer of the acrylate was not removed. The additives used—Bis-(4-tert-butylphenyl)iodonium hexafluorophosphate (Iod or SpeedCure 938) and ethyl-4-(dimethyl amino) benzoatewere (Omnirad EDB)—were obtained from Lambson Ltd. Wetherby, West Yorkshire, United Kingdom (UK).

### 3.3. Irradiation Sources

Two types of LEDs were used for the irradiation of samples: the first LED emits light at 385 nm with an intensity of 100 mW·cm^−2^ at the sample surface, and the second LED emits light at 405 nm with an intensity of 110 mW·cm^−2^ at the sample surface.

### 3.4. UV–Visible Absorption and Photolysis Experiments

We measured the UV–visible absorption spectra of PIs dissolved in acetonitrile using a JASCO V730 spectrometer (from Paris, France) which has a 1 cm light path. The concentration of PIs used in these experiments was 5 × 10^−5^ M. We conducted steady-state photolysis experiments using different formulations in acetonitrile which include the following: the PIs without any additive, PIs with iodine (10^−2^ M), and PIs with EDB (10^−2^ M). These formulations were exposed to an LED that emits light at 385 nm, and we measured their UV-vis spectra at different times using a JASCO V730 spectrometer (JASCO, Paris, France).

### 3.5. Photopolymerization Kinetics (RT-FTIR)

All polymerization experiments were carried out at room temperature with an LED@405 nm with irradiation beginning at time t = 10 s. The weight of the photoinitiating system was calculated from the monomer content (*w*/*w*). Overnight, the various photoinitiating systems and resins were mixed in glass bottles away from light exposure. Detailed experimental conditions for each formulation have been mentioned in the caption of figures. Using real-time Fourier transform infrared spectroscopy (JASCO FTIR 6600; from Paris, France), the conversion of the acrylate function of TA was continuously monitored. In order to decrease O_2_ inhibition, thin samples (~25 μm) were subjected to FRP of TA in laminate, where the formulations were sandwiched between two polypropylene films. From 1581 to 1662 cm^−1^, the C=C double bond band’s reduction was regularly monitored. The formulations were deposited within a 2.3 mm mold for the thicker samples (~2.3 mm in thickness). We continuously tracked the C=C band’s progression from 6117 to 6221 cm^−1^. The final acrylate function conversion (t = t_max_) of TA was calculated using Equation (1):

(1) $$FC\left(t\right)=\frac{A_{0}-A_{t}}{A_{0}}\times 100\%$$

where FC(t) is the function conversion at the time t, $A_{0}$ is the proportion of the peak area at 0 s, and $A_{t}$ is the portion of the peak area at t s. The procedure has been already described by our group in references [26,27].

### 3.6. Steady-State Fluorescence

The fluorescence characteristics of PIs in acetonitrile were measured using a JASCO FP-6200 spectrofluorometer (from Paris, France). Fluorescence quenching tests of PIs were performed using the JASCO FP-6200 spectrofluorimeter with successive additions of Iod or EDB (concentrations specified in the figure captions). PI concentrations in acetonitrile were 4 × 10^−5^ M during Iod addition and 5 × 10^−5^ M during EDB addition.

### 3.7. Oxidation and Reduction Potentials

The oxidation and reduction potentials (E_ox_ and E_red_) of PIs were assessed using cyclic voltammetry, with tetrabutylammonium hexafluorophosphate dissolved in acetonitrile as the electrolyte. Equation (2) was used to calculate the free energy change (ΔG_et_) for the electron transfer processes of PIs with both Iod and EDB [29]. The symbols E_ox_, E_red_, E*, and C represent the electron donor’s oxidation potential, the electron acceptor’s reduction potential, the energy level of the excited state under consideration, and the coulombic term for the first-formed ion pair, respectively. In this case, C was disregarded as is customary for polar solvents, the reduction potential of Iod is E_red_(Iod) = −0.7 V, and the oxidation potential of EDB is E_ox_(EDB) = 1 V [29].

ΔG_et_ = E_ox_ − E_red_ − E* + C (2)

### 3.8. ESR Spin-Trapping (ESR-ST) Experiments

The ESR-ST experiments were carried out using an X-Band spectrometer (Bruker EMX-plus; from Karlsruhe, Germany). The experiments were conducted at room temperature (RT) under N_2_ with a 405 nm LED irradiation within the ESR spectrometer’s cavity. Using phenyl-N-tert-butylnitrone (PBN) from TCI-Europe, radicals were trapped in tert-butylbenzene using a method outlined in references [26,29]. The ESR spectrum simulations were obtained using the PEST WINSIM program.

### 3.9. Direct Laser Writing Experiments

A laser diode at 405 nm was employed for the spatially controlled irradiation in the direct laser writing experiments. The laser’s spot size was 50 μm, and its intensity was 110 mW. The photopolymerization process was conducted under air, as documented in the literature [29]. The resin formulation was deposited on and between microscopic slides. The obtained three-dimensional patterns were well fixed on the microscopic slides where ethanol was used to remove the non-polymerized solution, they were not subjected to a post treatment, and they were analyzed using a numerical optical microscope (DSX-HRSU from Olympus Corporation, Tokyo, Japan).

## 4. Conclusions

In this article, two new photoinitiators based on the acridone molecule were successfully synthesized and used as moderate/high-performance visible light photoinitiators for the free radical polymerization of acrylates upon irradiation with LED@405 nm. Photoinitiation mechanisms for these molecules acting as one-component Type II photoinitiators and as multicomponent photoinitiating systems in the presence of an iodonium salt or an amine synergist (EDB) were established by examining the photochemical properties utilizing steady-state photolysis experiments, fluorescence quenching experiments, cyclic voltammetry, and through the ESR spin-trapping experiments. Remarkably, the high photoreactivity of the photoinitiating system (DPM-Acr/Iod) allowed the fabrication of 3D patterns by laser writing technologies. Forthcoming innovations on the acridone molecule to be used as highly efficient photoinitiators at longer wavelengths and for 3D printing applications will be suggested in upcoming studies.

## Figures and Tables

**Table 1:** Light absorption properties of the investigated compounds: maximum absorption wavelength (λ_max_) and molar extinction coefficients at λ_max_ and at 405 nm.

PIs	λ_max_ (nm)	ε_max_ (M^−1^cm^−1^)	ε_405_ (M^−1^ cm^−1^)
Bn-Acr	395	920	2600
	376	6900	
DPM-Acr	392	8100	2000
	375	6300	

**Table 2:** FCs of acrylate function using PIs (1%*w*/*w*), PIs/Iod (1%/1% *w*/*w*), PIs/EDB (1%/1% *w*/*w*), Iod alone (1% *w*/*w*), and EDB alone (1% *w*/*w*) after 200 s of irradiation with an LED (λ = 405 nm).

PI Systems	Thin Samples (25 μm) in Laminate @405 nm
Bn-Acr	69%
DPM-Acr	68%
Bn-Acr/Iod	77%
DPM-Acr/Iod	81%
Bn-Acr/EDB	70%
DPM-Acr/EDB	60%
Iod	26%
EDB	19%

**Table 3:** Parameters characterizing the chemical mechanisms associated with the PI/Iod and PI/EDB interactions in acetonitrile.

	E_S1_ (eV)	E_ox_ (V)	ΔG_S1(PI/Iod)_ (eV) ^a^	E_red_ (V)	ΔG_S1(PI/EDB)_ (eV) ^b^	K_sv (Iod)_ (M^−1^) ^c^	Φ_et (PI/Iod)_^d^	K_sv (EDB)_ (M^−1^) ^c^	Φ_et (PI/EDB)_^d^
Bn-Acr	3.05	1.4	−0.95	−1.2	−0.85	56	0.76	40	0.8
DPM-Acr	3.08	1.46	−0.92	Not present		35	0.68	30	0.74

a: Evaluated from ∆G_S1_ (PI/Iod) = E_ox_ − E_red_(Iod) − E_S1_; E_red_(Iod) = −0.7 V. b: Evaluated from ∆G_S1_ (PI/EDB) = E_ox_(EDB) − E_red_ − E_S1_; E_ox_ (EDB) = 1 V. c: Stern–Volmer coefficient (Ksv): the slope of the quenching curve (I_0_/I = 1 + K_sv_[Iod]). d: Electron transfer quantum yield (Φ_et_): obtained according to the following equation: Φ_et_ = ksv[Iod]/(1 + ksv[Iod]).

## Data Availability

The data underlying this study are available in the published article and its Supporting Information.

## Supplementary Materials

The following supporting information can be downloaded at: https://www.mdpi.com/article/10.3390/molecules29194715/s1, Figure S1: photopolymerization profiles of TA under air; Figure S2: Photolysis of PIs with EDB; Figure S3: Stern-Volmer plots; Figure S4: Cyclic voltammetry of PIs; Figure S5: ESR spectra for Bn-Acr/EDB; Synthesis of the investigated acridone-based PIs with their characterization tests.
