# Supplementary material for: Acridone Derivatives for Near-UV Radical Polymerization: One-Component Type II vs. Multicomponent Behaviors
Source: Molecules. 2024 Oct 5;29(19):4715. doi: 10.3390/molecules29194715 (PMC11478236; doi:10.3390/molecules29194715)

Supporting Information

Acridone derivatives for near-UV radical polymerization: one-component Type II vs. multicomponent behaviors

Adel Noon[1,2,3], Francesco Calogero[4,5], Andrea Gualandi,[4,5] Hiba Hammoud[1,2], Tayssir Hamieh[3,6], Joumana Toufaily[3], Fabrice Morlet-Savary[1,2], Michael Schmitt[1,2], Pier Giorgio Cozzi[4,5*], Jacques Lalevée[1,2*]

[1] Université de Haute-Alsace, CNRS, IS2M UMR 7361, F-68100 Mulhouse, France.

[2] Université de Strasbourg, Strasbourg, France.

[3] Laboratory of Materials, Catalysis, Environment and Analytical Methods (MCEMA), Faculty of Sciences, Doctoral School of Sciences and Technology (EDST), Lebanese University, Beirut 6573-14, Lebanon.

[4] Department of Chemistry Giacomo Ciamician, University of Bologna, Bologna, Italy.

[5] Center for Chemical Catalysis-C3, Alma Mater Studiorum-Università di Bologna, Via Selmi 2, 40126 Bologna, Italy.

[6] Faculty of Science and Engineering, Maastricht University, P.O. Box 616, 6200 MD Maastricht, Netherlands.

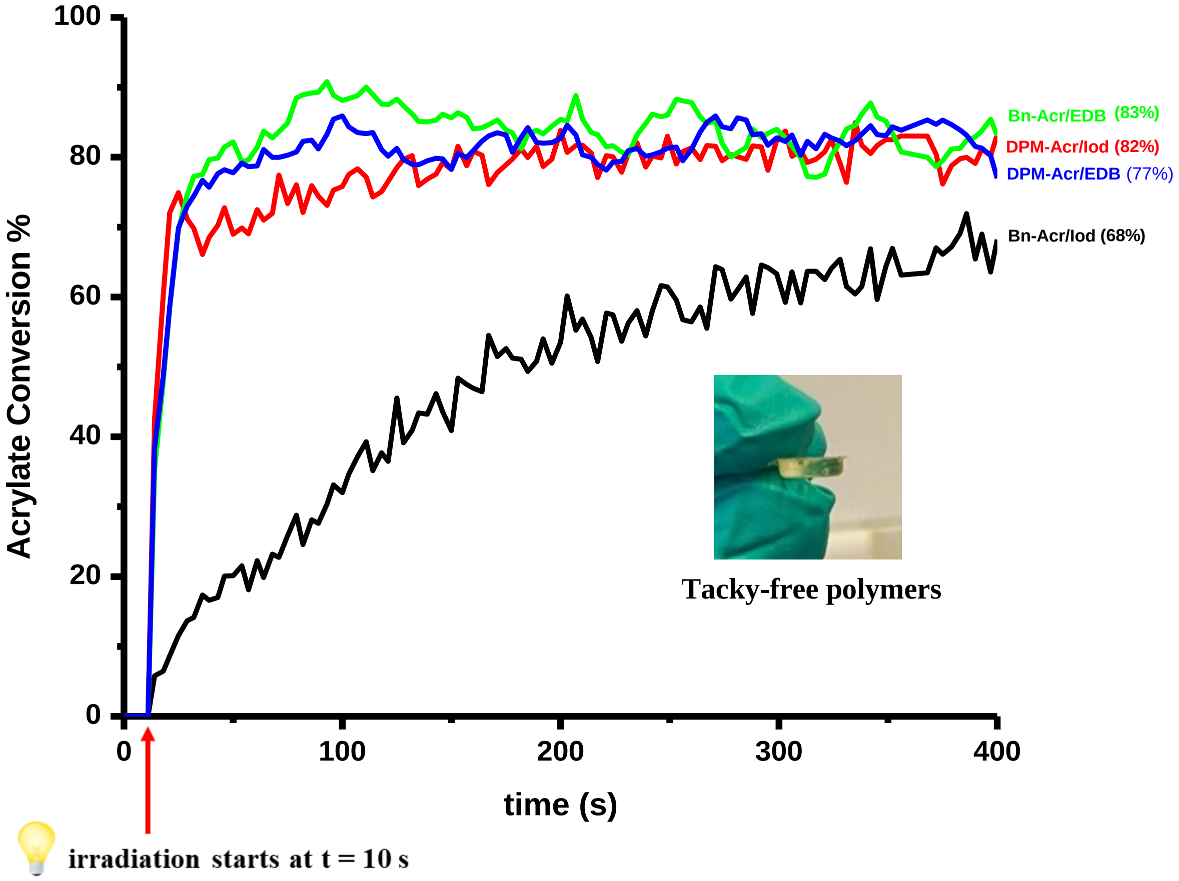

Figure S1. Photopolymerization profiles of TA (acrylate function conversion vs. irradiation time) under air (thickness = 2.3 mm) upon exposure to a LED (λ = 405 nm) in the presence of PIs/Iod (1%/1% w/w), and PIs/EDB (1%/1% w/w). The irradiation starts at t = 10 s.

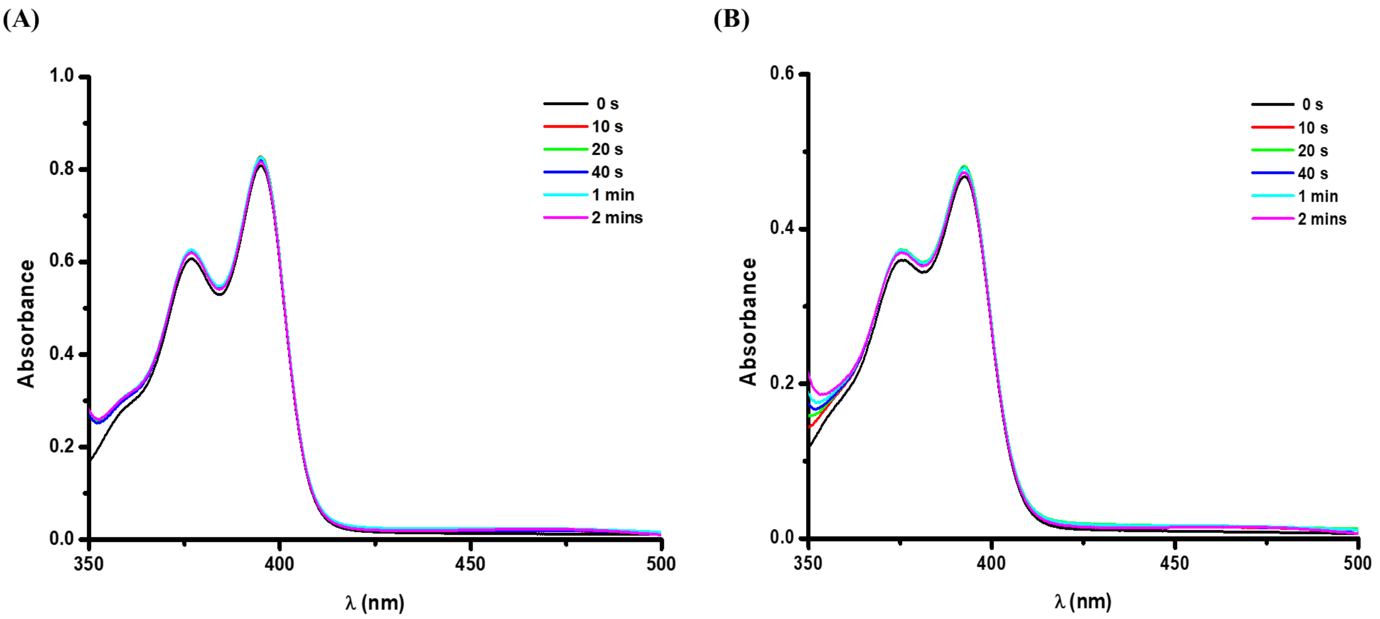

Figure S2. Photolysis of (A) **Bn-Acr**, and (B) **DPM-Acr** with EDB (10^{-2} M) in acetonitrile using LED at λ = 385 nm.

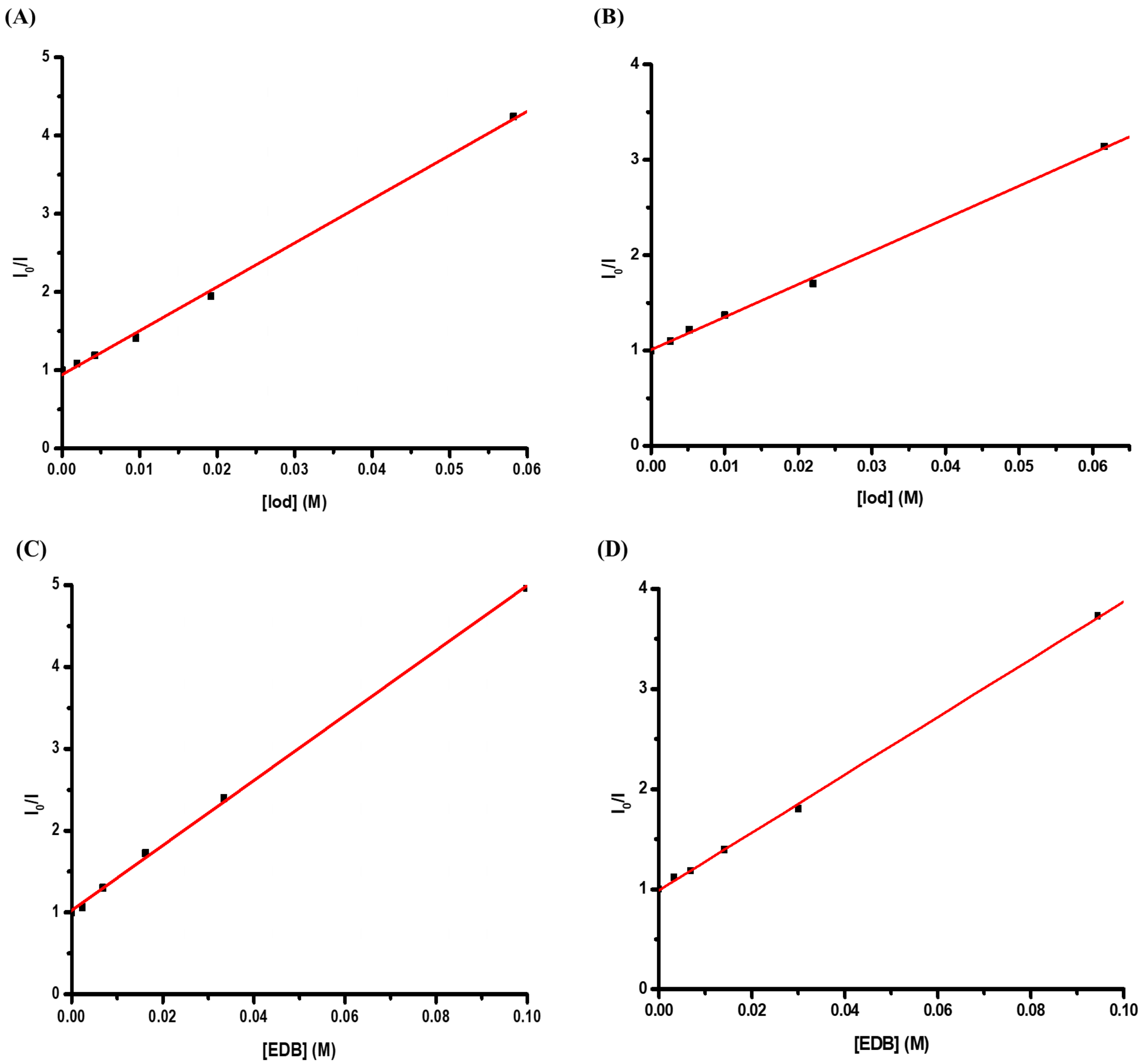

Figure S3. Stern-Volmer plots associated to the fluorescence quenching experiments of **Bn-Acr** (A), and **DPM-Acr** (B) by Iod in acetonitrile; fluorescence quenching experiments of **Bn-Acr** (C), and **DPM-Acr** (D) by EDB in acetonitrile.

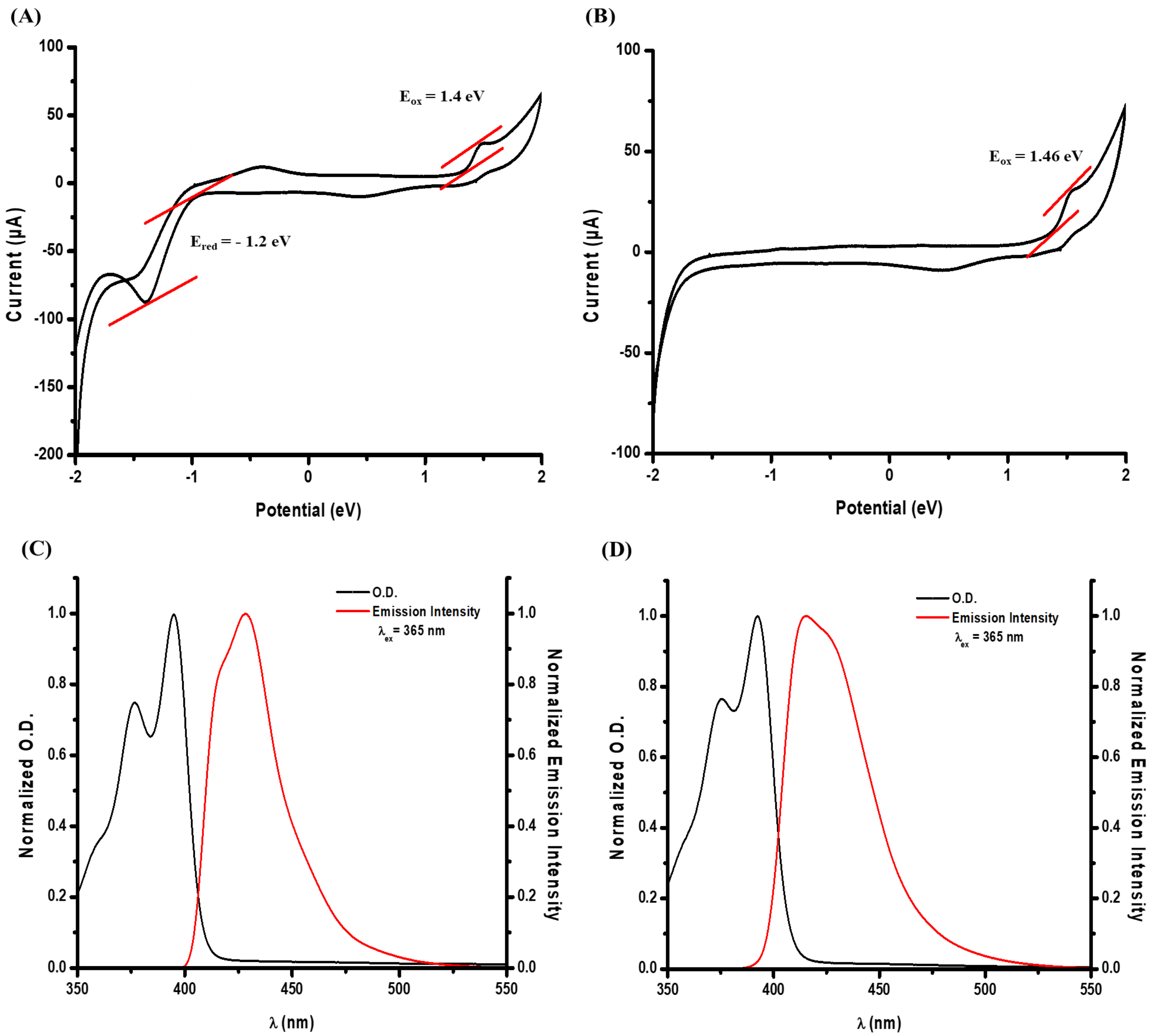

Figure S4. Cyclic voltammetry of **Bn-Acr** (A), and **DPM-Acr** (B) using tetrabutylammonium hexafluorophosphate dissolved in acetonitrile as the electrolyte; UV-visible absorption and emission spectra of **Bn-Acr** (C), and **DPM-Acr** (D) in acetonitrile.

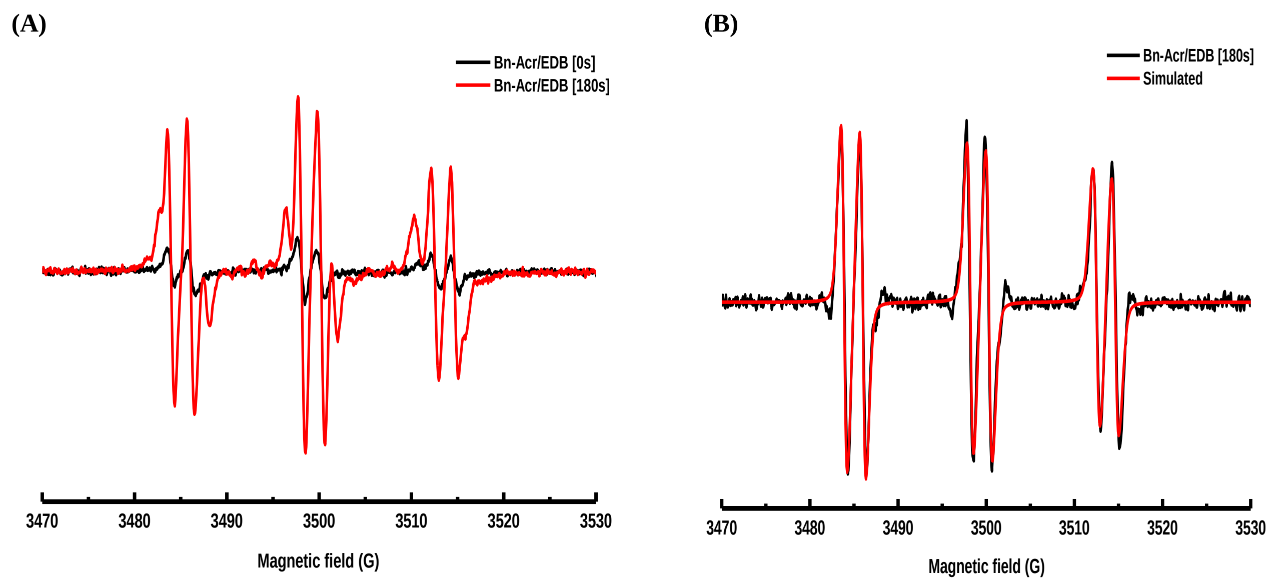

Figure S5. ESR spectra for **Bn-Acr**/EDB recorded in the presence of PBN and *tert*-butylbenzene with a LED@405nm: (A) before and after irradiation; (B) experimental and simulated spectra observed after irradiation (at t = 180 s).

Synthesis of the investigated acridone-based PIs

General information

1H-NMR spectra were recorded on Varian Mercury 400 or Bruker 600 spectrometers. Chemical shifts are reported in ppm from TMS with the solvent resonance as the internal standard ($CHCl_3$: δ = 7.26 ppm). Data are reported as follows: chemical shift, multiplicity (s = singlet, d = duplet, t = triplet, q = quartet, dd = double duplet, m = multiplet), coupling constants (Hz). ^{13}C-NMR spectra were recorded on Varian Mercury 400 or Inova 600 or Bruker 600 spectrometers. Chemical shifts are reported in ppm from TMS with the solvent as the internal standard ($CDCl_3$: δ = 77.0 ppm).

HRMS were performed on Waters Xevo G2-XS QTof, ESI+, cone voltage 40 V, Capillary 3KV, source temperature 120 °C.

ATR-FTIR spectra of pure compounds were recorded with a Bruker Alpha instrument in transmittance mode with a 4 cm^{-1} resolution in the 4000-400 cm^{-1} range.

All the reagents were purchased from commercial sources (Sigma-Aldrich, Alfa Aesar, Fluorochem, Strem Chemicals, TCI) and used without further purification unless specified.

All reactions requiring inert atmosphere were set up under an argon in heat gun-dried glassware using standard Schlenk techniques unless specified.

Anhydrous solvents were supplied by Aldrich in Sureseal® bottles and, unless specified, were used without further treatment.

Synthesis of Bn-Acr

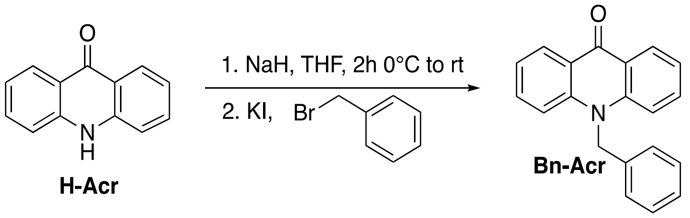

10-benzylacridin-9(10*H*)-one
Chemical Formula: $C_{20}H_{15}NO$
Molecular Weight: 285,35

In a vacuum-flamed 100 mL double-necked flask, acridone (H- Acr 390 mg, 2 mmol, 1 equiv.) was added, under an inert nitrogen atmosphere, to anhydrous DMF (20 mL). The solution was placed at 0°C in an ice and water bath for the addition of NaH (60% in oil, 196 mg, 2.4 mmol, 1.2 equiv.). After stirring at rt for approximately 2 hours, BnBr (0.36 mL, 3 mmol, 1.5 equiv.) and KI (66.4 mg, 0.4 mml, 0.2 equiv.) were added to the solution. After stirring overnight, the solution was quenched with H_2O until a light yellow solid precipitated. Afterwards, vacuum filtration was performed with subsequent recrystallization with $CHCl_3$/Ethanol. The pure product Bn-Acr was isolated with a yield of 54%. ^{1}H NMR (600 MHz, $CDCl_3$) δ 8.61 (d, J = 8.0 Hz, 2H), 7.67 – 7.61 (m, 2H), 7.39 – 7.28 (m,

7H), 7.22 (d, J = 7.5 Hz, 2H), 5.61 (s, 1H). ^{13}C NMR (151 MHz, $CDCl_3$) δ 178.64, 142.97, 135.86, 134.43, 129.62, 128.19, 126.02, 123.00, 122.02, 115.55, 51.21. HRMS *m/z* = $[M+H]^+$ calculated for $C_{20}H_{16}NO^+$ 286.1226, found 286.1232. ATR-FTIR: *ν* (cm^{-1}) 1629, 1593, 1459, 1488, 1378, 1366, 1339, 1263, 1205, 1176, 1104, 1042, 1031, 934, 858, 801, 749, 596.

Synthesis of S1

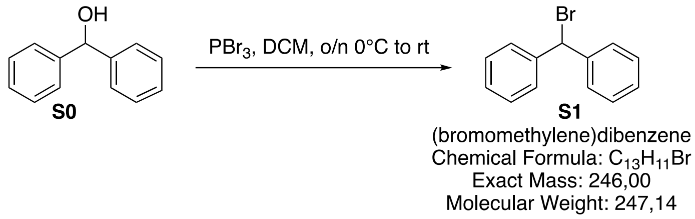

1,1-diphenylmethanol (S0, 920 mg, 5 mmol, 1 equiv.) and dry DCM (10 mL) were added to a vacuum-flamed 50 mL two-necked flask, under an inert nitrogen atmosphere. The solution was brought to 0°C using an ice and water bath for the dropwise addition of PBr_3 (0.3 mL, 3 mmol, 0.6 equiv.). After reporting to rt, he was left agitated all night. After quenching with water and extracting three times with Hexane and NaHCO3, the organic phases were combined and dried with Na2SO4 and subsequently filtered. The organic solvents were removed under vacuum. The reaction crude was purified by chromatographic column (Cyclohexane/EtOAc) thus obtaining the desired product S1 as a light brown crystalline solid with a yield of 46%. Spectroscopical data are in accordance with the reported literature. https://doi.org/10.1039/C5OB02598B

Synthesis of DPM-Acr

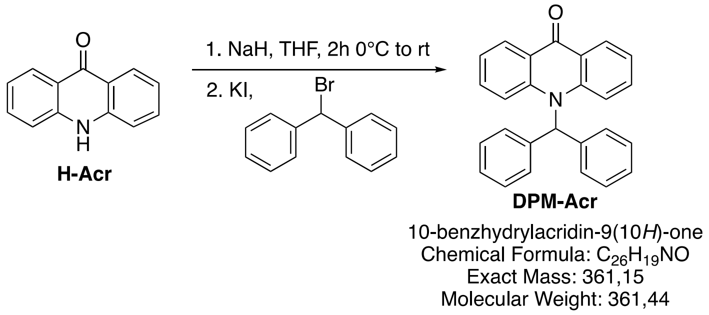

In a vacuum-flamed 50 mL two-neck flask, acridone (H- Acr 195 mg, 1 mmol, 1 equiv.) was added, under an inert atmosphere, to anhydrous DMF (10 mL). The solution was placed at 0 °C in an ice and water bath for the addition of NaH (60% in oil, 48 mg, 1.2 mmol, 1.2 equiv.). After stirring at rt for approximately 2 hours, diphenylbromomethane (370 mg, 1.5 mmol, 1.5 equiv.) and KI (33.2 mg, 0.2 mml, 0.2 equiv.) were added to the solution. After stirring overnight, the solution was quenched with H2O until a precipitate was formed which was filtered and purified using a chromatographic column

(10% EtOAcin Hexane) giving the desired product with a yield of 24%. ^{1}H NMR (600 MHz, $CDCl_3$) δ 8.52 (dd, J = 8.0, 1.8 Hz, 2H), 7.43 – 7.38 (m, 2H), 7.38 – 7.30 (m, 7H), 7.29 – 7.27 (m, 6H), 7.24 – 7.17 (m, 2H). 13C NMR (151 MHz, CDCl3) δ 179.01, 143.24, 137.76, 132.93, 128.95, 128.32, 128.10, 127.46, 123.87, 121.76, 118.04, 67.58. HRMS *m/z* = $[M+H]^+$ calculated for $C_{26}H_{20}NO^+$ 362.1539, found 362.1544. ATR-FTIR: ν (cm^{-1}) 1631, 1590, 1453, 1486, 1378, 1366, 1342, 1260, 1200, 1171, 1112, 1041, 1035, 935, 850, 800, 750, 595.

NMR Traces.

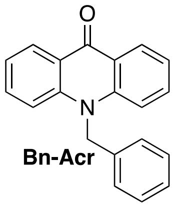

10-benzylacridin-9(10*H*)-one
Chemical Formula: $C_{20}H_{15}NO$
Molecular Weight: 285,35

^{1}H NMR (600 MHz, $CDCl_3$)

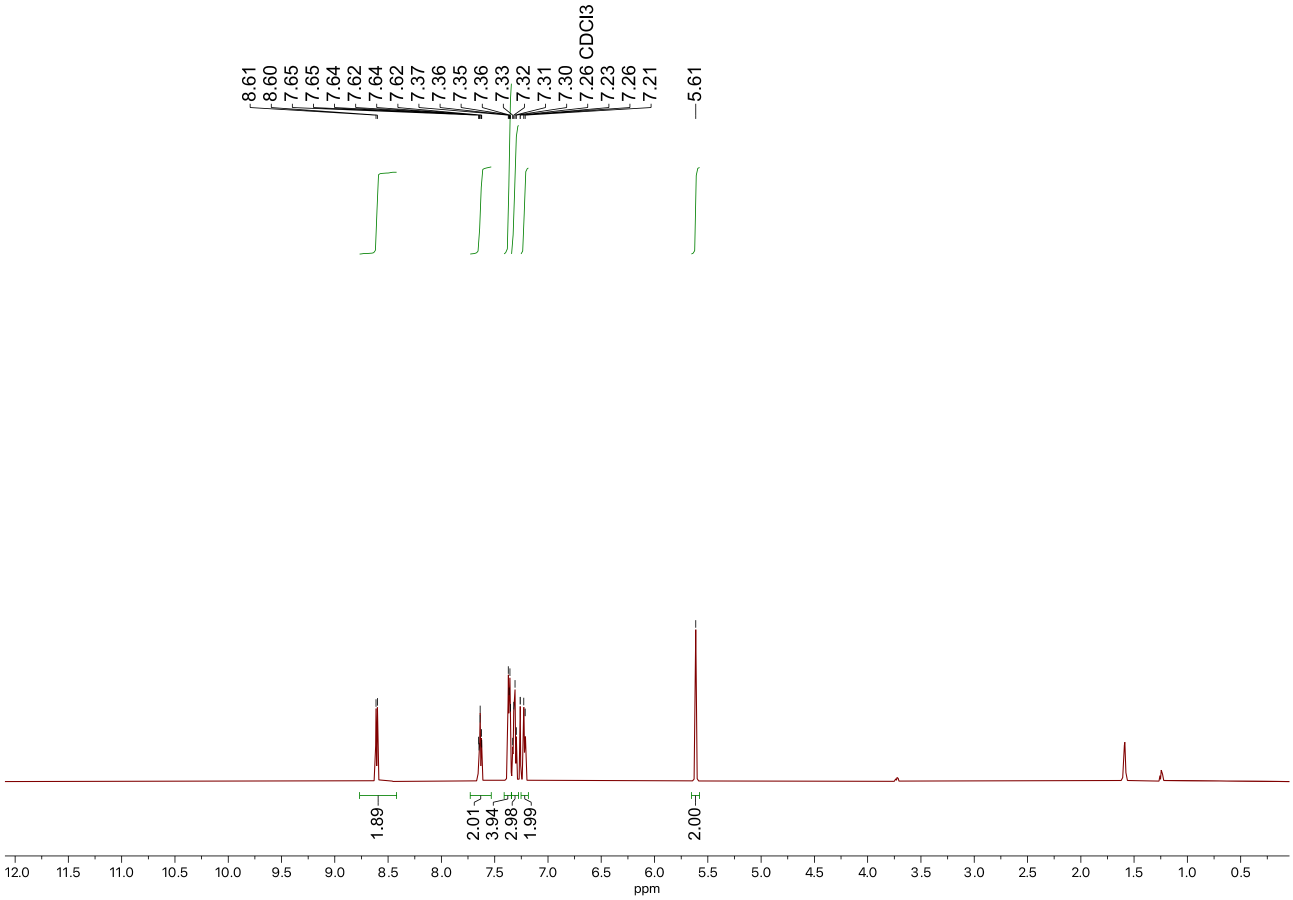

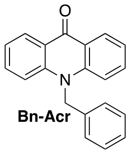

10-benzylacridin-9(10*H*)-one
Chemical Formula: $C_{20}H_{15}NO$
Molecular Weight: 285,35

$^{13}C\{^{1}H\}$ NMR (150 MHz, $CDCl_3$)

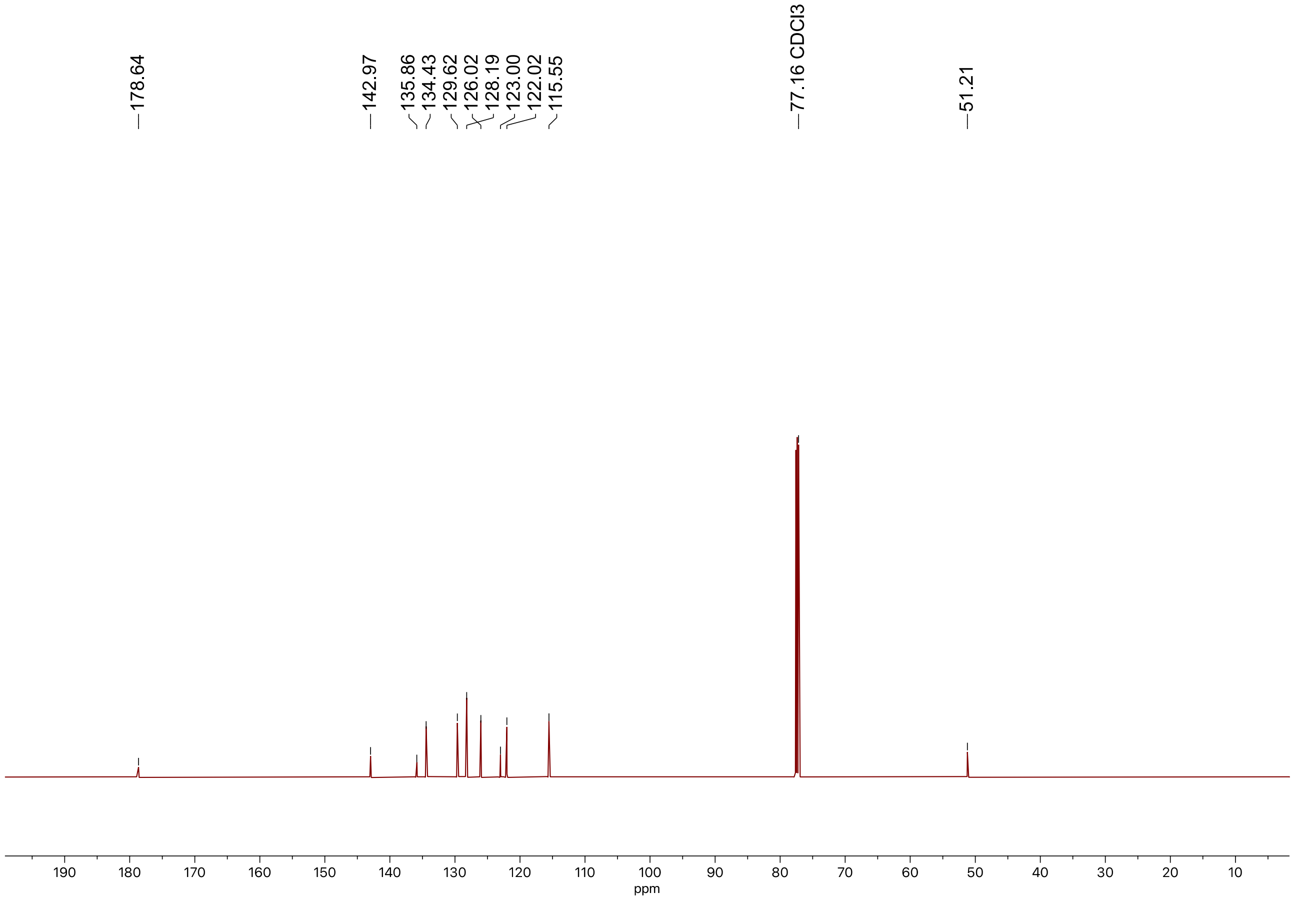

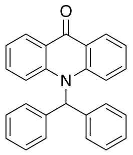

DPM-Acr

10-benzhydrylacridin-9(10*H*)-one
Chemical Formula: $C_{26}H_{19}NO$
Exact Mass: 361,15
Molecular Weight: 361,44

^{1}H NMR (600 MHz, $CDCl_3$)

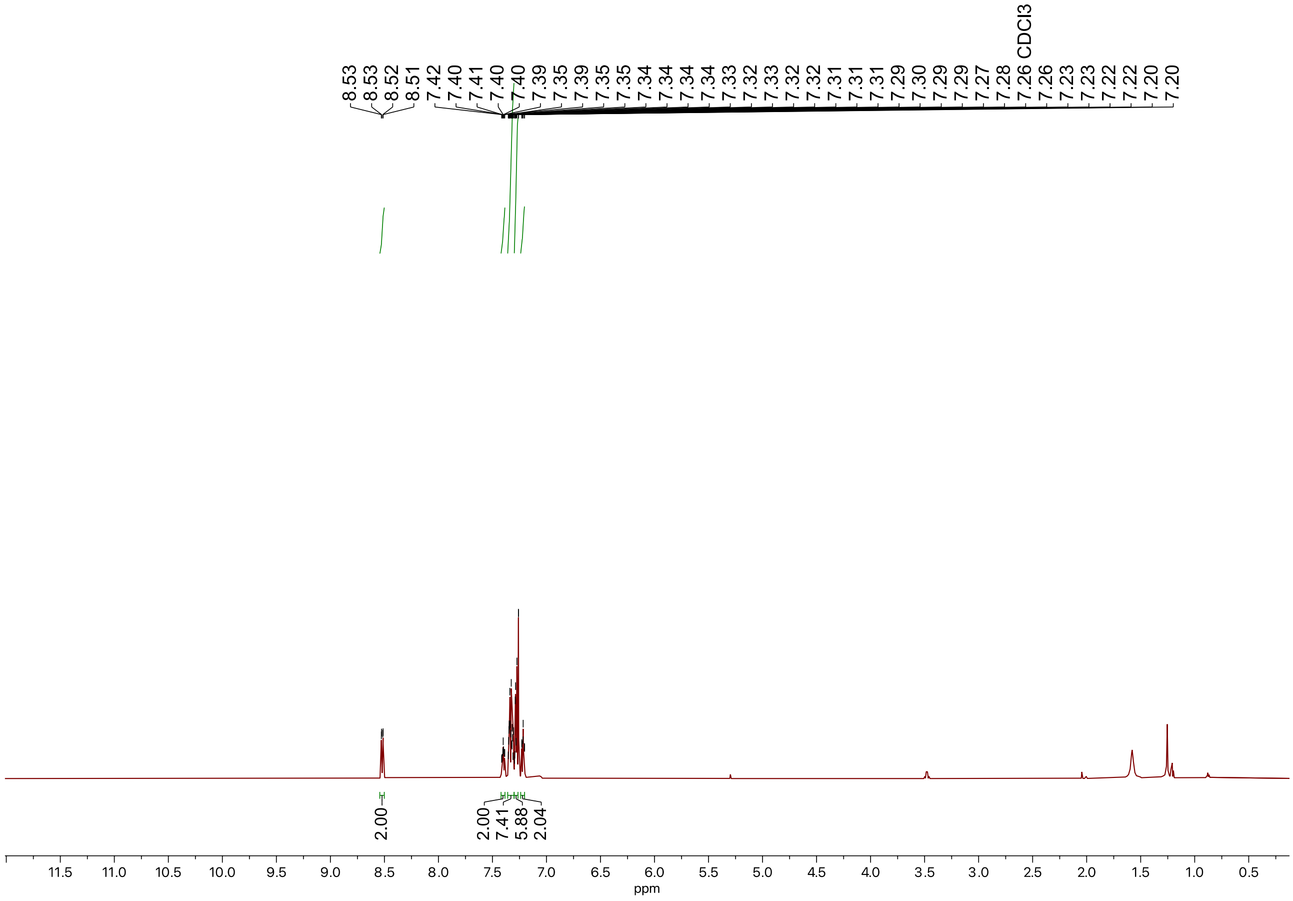

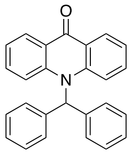

DPM-Acr

10-benzhydrylacridin-9(10*H*)-one
Chemical Formula: $C_{26}H_{19}NO$
Exact Mass: 361,15
Molecular Weight: 361,44

^{13}C{^{1}H} NMR (150 MHz, $CDCl_3$)

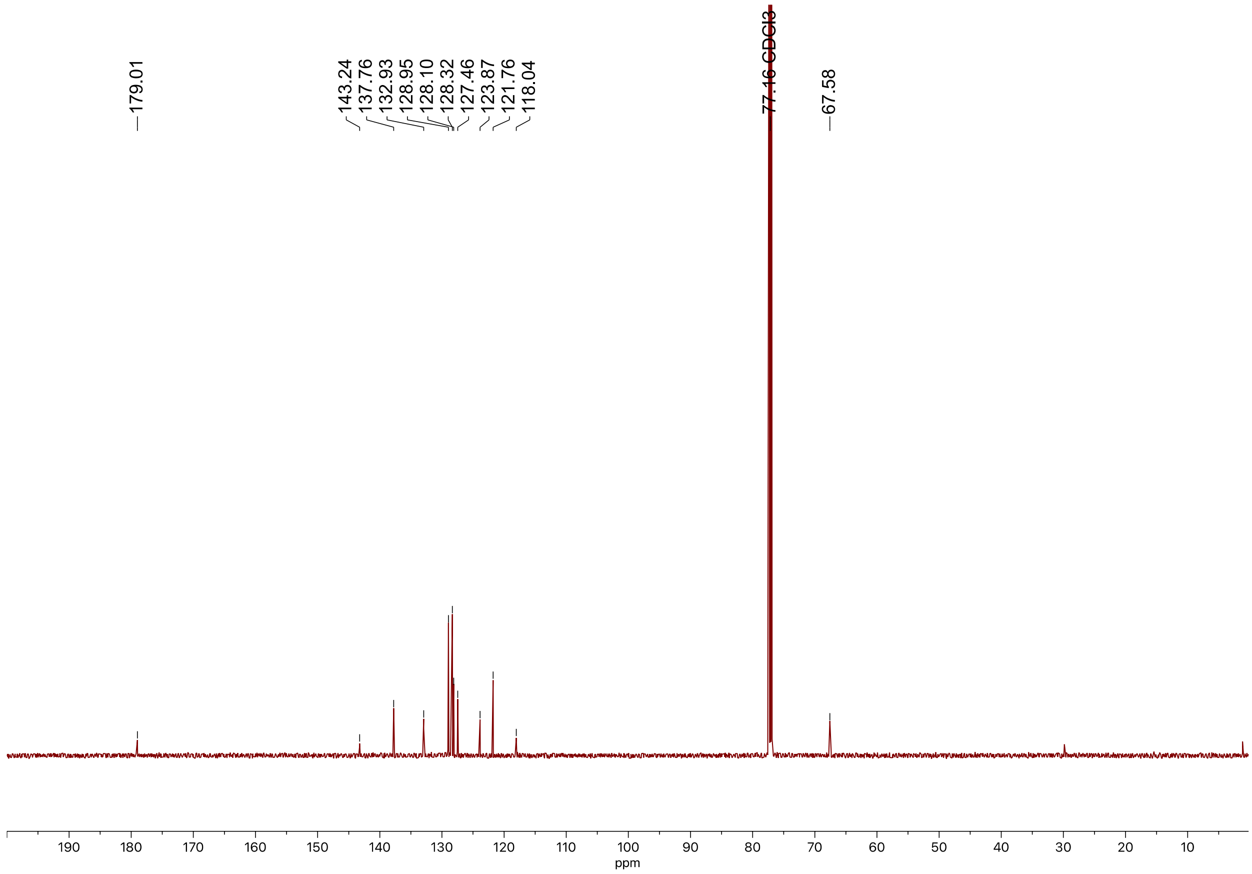

Supplement: Supplementary file 1 [file molecules-29-04715-s001.zip › molecules-3211023-supplementary.pdf]
